# Using context-specific effect of miRNAs to identify functional associations between miRNAs and gene signatures

**DOI:** 10.1186/1471-2105-14-S12-S1

**Published:** 2013-09-24

**Authors:** Mohammed Alshalalfa, Reda Alhajj

**Affiliations:** 1 Department of Computer Science, University of Calgary, Calgary, Alberta, Canada; 2 Biotechnology Research Center, Palestine Polytechnic University, Hebron, Palestine; 3 Computer Science Department, Global University, Beirut, Lebanon

## Abstract

**Background:**

MicroRNAs are a class of short regulatory RNAs that act as post-transcriptional fine-tune regulators of a large host of genes that play key roles in many cellular processes and signaling pathways. A useful step for understanding their functional role is characterizing their influence on the protein context of the targets. Using miRNA context-specific influence as a functional signature is promising to identify functional associations between miRNAs and other gene signatures, and thus advance our understanding of miRNA mode of action.

**Results:**

In the current study we utilized the power of regularized regression models to construct functional associations between gene signatures. Genes that are influenced by miRNAs directly(computational miRNA target prediction) or indirectly (protein partners of direct targets) are defined as functional miRNA gene signature. The combined direct and indirect miRNA influence is defined as context-specific effects of miRNAs, and is used to identify regulatory effects of miRNAs on curated gene signatures. Elastic-net regression was used to build functional associations between context-specific effect of miRNAs and other gene signatures (disease, pathway signatures) by identifying miRNAs whose targets are enriched in gene lists. As a proof of concept, elastic-net regression was applied on lists of genes downregulated upon pre-miRNA transfection, and successfully identified the treated miRNA. This model was then extended to construct functional relationships between miRNAs and disease and pathway gene lists. Integrating context-specific effects of miRNAs on a protein network reveals more significant miRNA enrichment in prostate gene signatures compared to miRNA direct targets. The model identified novel list of miRNAs that are associated with prostate clinical variables.

**Conclusions:**

Elastic-net regression is used as a model to construct functional associations between miRNA signatures and other gene signatures. Defining miRNA context-specific functional gene signature by integrating the downstream effect of miRNAs demonstrates better performance compared to the miRNA signature alone (direct targets). miRNA functional signatures can greatly facilitate miRNA research to uncover new functional associations between miRNAs and diseases, drugs or pathways.

## Background

MicroRNA(miRNA)-mediated regulation constitutes a new dimension of gene expression regulation research [1-3]. MiRNA are short (18-24) nt non-coding RNA class that has played a critical regulatory role to fine-tune gene expression in wide range of biological processes. Since their discovery [4], they emerged as a significant regulatory layer of gene regulation at the post-transcriptional level. MiRNAs bind to the 3'UTR of genes and cause destabilization or translational repression of target mRNAs in a mechanism that is not fully understood. More than 50% of the human protein-coding genes are regulated by miRNAs [5]; each miRNA targets hundreds of genes which makes them critical molecules that deserve considerable amount of research. Several biological processes ranging from cell differentiation to metabolism are regulated by miRNA [3]. Additionally, a growing list of diseases [6,7], like cancer, biological pathways, molecular concepts, are associated with miRNAs. For example, miRNA-1, miRNA-16, miRNA-143, and many others are very important miRNAs that have significant impact on prostate cancer development [8-10].

The current major challenge in miRNA research is characterizing miRNA mode of action and determining the pathways and diseases they are involved in. Determining the role of individual miRNAs in cellular regulatory processes is still a major challenge. The function of many miRNAs remains unknown, and even for relatively well studied miRNAs, only a handful of their targets have been characterized [11,12]. Characterizing the functions of miRNA targets reveals higher level of understanding of the miRNA function. Thus one of the key steps in genomic studies is to infer miRNAs that target the genes of interest. Identifying and characterizing reference biological concepts, for example miRNA targets, overrepresented in a list of genes that results from biological experiments is a powerful methodology to characterize the function hidden in the gene list. This area of research which is also known as gene enrichment analysis has gained a considerable body of research. Several tools, such as DAVID [13] and GeneMANIA [14] that employ the available gene annotations have been developed to identify the enriched gene annotations (GO, pathways) in a list of genes of particular interest, Geneset2miRNA [15] and Expression2kinases [16] are used to discover enriched miRNAs in gene sets. A comprehensive comparison among 68 enrichment tools [17] identified three major trends in enrichment analysis; namely, Gene Set Enrichment Analysis(GSEA) [18], Over Representation Analysis(ORA) and Modular Enrichment Analysis(MEA). Most of the 68 tools belong to the second group as they use statistical tests like fisher and hypergeometric tests to assess the overrepresentation of particular term. Though these tools are well established as standard tools for enrichment analysis, we find these tools lack modular concept of gene lists. Integrating the interactions between gene sets to assess the overrepresentation is a promising direction to follow to gain system level understanding of gene enrichment analysis.

Since the cell is a complex system of interacting genes, proteins, miRNA and other macromolecules, incorporating biological networks is valuable modeling structure to define network-based functional similarity measures between genes signatures Constructing functional associations between gene sets (signatures) helps to reveal the underlying biological mechanism linking the gene sets. Building functional associations between diseases and pathways uncovers the dysregulated pathways in complex diseases like cancer. Taking this into consideration, inferring the miRNA function from the downstream or upstream biological context is effective and has revealed novel miRNA functions. Integrating the protein context of miRNA targets is a promising dimension for miRNA function prediction and for linking miRNAs to pathways and diseases. Protein-network based functional enrichment analysis is a new trend in enrichment analysis. Several studies started incorporating the network topology of the gene sets [19-22]. One way to incorporate network in enrichment is to extend the gene sets by incorporating the protein neighbors of the genes sets and then apply standard enrichment tests like Fisher's and hypergeometric tests [19]. Another track is to assess the connectedness of the overlapped genes; more interconnected gene sets indicate more functional association [21].

In the past few years, the functional association between miRNAs and protein interactions gained a significant body of attention. Here we use the term context-specific miRNA effect to represent the effect of miRNA the partners of the miRNA targets in the protein network. Though miRNAs target a wide range of genes that play role in most of the biological processes, analyzing the characteristics of the targets in protein networks showed that there is a significant correlation between the protein degree in protein networks and the number of targeting miRNAs, highly connected proteins are controlled by larger number of miRNAs [23]. This functional property between topological features of biological networks has been employed to reduce noise in discovering miRNA-mRNA interactions [24]. Single miRNAs and miRNA custers showed to target multiple protein members of single protein complexes [25]. For example, SMAD3-SMAD4-FOXO3 complex is enriched with miR-1284 targets, and MAD1-SIN3A-HDAC2 complex is enriched with targets of the miR-510-514 and miR-1912-1264 clusters. Other studies demonstrated that the targets of miRNAs are modular; the targets of particular miRNA are interacting in protein networks, thus considering the miRNA context-specific effect provides higher level of understanding of miRNA function when compared to employing only direct targets of miRNAs [23]. Previously [26], we showed that using the indirect targets of miRNA to represent the miRNA gene signature is effective to reveal the treating miRNAs from a set of downregulated genes upon pre-miRNA treatment. The previous study showed a proof of concept that integrating protein networks to form context-specific miRNA effect is informative to identify miRNA mode of action. Our previous analysis suggests that integrating functional protein networks to functionally characterize miRNA function helps researchers to gain system-level understanding of the gene list of interest. To the best of our knowledge, no protein network-based method has been developed particularly for miRNA enrichment analysis.

The goal of this work is use interactions among protein when assessing the overrepresentation of miRNA targets in a set of genes. This would lead us to build functional associations between gene sets, for example, miRNA targets and disease signatures. For this purpose we used regularized regression model to predict influence coefficient of miRNAs on disease signatures. The resulted coefficients are used to reveal miRNA enrichment in the gene set. This approach is applied to uncover functional associations between miRNAs, disease and pathways. This work will advance our understanding of the mode of action of miRNAs and their influence on the context of the targets. This model can be applied to associate gene signatures in general. In this work we only focused on miRNA gene signatures.

## Materials and methods

### Biological interactions

For the course of this study we used two sets of miRNA-target interactions. The firs is computationally predicted miRNA targets downloaded from TargetScan [27][PredNet], and the second is experimentally validated miRNAs and their targets that were extracted from two public databases mirTarbase [28] and miRecord [29]. The union of mirTarbase and miRecord was used as a source of experimentally validated miRNA-target interactions [ExpNet]. For protein networks, we used undirected functional protein interactions from Reactome database [30]. The protein networks are used in conjugation with the miRNA target networks to find the partners of each miRNA targets.

### Defining context-specific effect of miRNA signature

In this section we first explain how the context-specific effect of miRNAs is constructed from miRNA-targets networks and protein networks. miRNA(*miR*) binds to a mRNA(*m*) directly by binding to the miRNA response element (MRE) in the 3'UTR or indirectly by influencing a PPIN neighbor of the direct targets. The direct miRNA-target interactions are used from PredNet, and the indirect interactions, which we will refer to in this work as miRNA context-specific effect, is built by integrating miRNA-targets with PPIN. The constructed miRNA context-specific effect (*miRNet*) was constructed as described in our previous work [26].

### Model performance assessment

In this work we hypothesize that given a gene signature (list of gene of particular interest), we can predict the enriched miRNAs in the gene signature using the proposed *miRNet *miRNA-target interactions. First, we used seven gene sets that were retrieved from public data sets with known enriched miRNA. This list of genes are genes that are downregulated upon miRNA transfection to HeLa and LNCaP cell and are used to demonstrate a proof-of-concept of the proposed model. The lists are described in Table 1. A prostate cancer signature is identified from MSKCC Prostate cancer cohort(GSE21032); 480 genes were identified as down regulated in prostate cancer compared to normal samples, and 51 as upregulated using Significant Analysis of Microarays(SAM) [31].

**Table 1:** Summary of gene lists used in this study to validate the performance of the proposed method in comparison with existing algorithms

Experiment description	Enriched miRNAs	Number of reported genes	Reference
*HeLa cells transfected with miR-1*	*miR-1*	*96 repressed genes*	[35]
*HeLa cells transfected with miR-124*	*miR-124*	*174 repressed genes*	[35]
*HeLa cells transfected with miR-373*	*miR-373*	*65 repressed genes*	[35]
*LNCaP cells treatedwith pre-miR-1*	*miR-1*	*88 repressed genes and 80* *upregulated genes*	[36]
*LNCaP cells treatedwith pre-miR-206*	*miR-206*	*83 repressed genes and 62* *upregulated genes*	[36]
*LNCaP cells treatedwith pre-miR-27b*	*miR-27b*	*51 repressed genes and 157* *upregulated genes*	[36]
*LNCaP cells transfected with pre-miR-32*	*miR-32*	*67 repressed genes*	[37]
*LNCaP cells transfected with pre-miR-148a*	*miR-148a*	*79 repressed genes*	[37]
*Genes predicted to be targets of 11* *prostate miRNAs extracted from PRedNet*	*miR(1, 204*, *205, 143, 145*, *221, 222*, *27b, 133b, 31*, *let-7*	*1854*	[5,12,38]
*Altered genes in prostate cancer using* *Taylor data*		*480 downregulated in prostate and 51 upregulated in prostate 269 downregulated genes in PCa samples that have BCR recurrence*	[39]
*Genes associated with BCR event in * *prostate cancer*			[39]

### Predicting miRNA influence coefficient using regularized regression to build functional association

In this section we explain how we used regularized regression model to find miRNAs enriched in gene lists. The model takes two inputs; miRNA-target(gene) interactions and a gene list. We used PredNet and miRNet separately to determine the initial variables (miRNAs) in the regression model; each variable represents the influence of a miRNA on all targets in miRNA-target interactions. We used the genes downregulated upon miRNA treatment as response variable (*GeneSignature*) in the regression model. The regularized regression model predicts non-redundant miRNAs that influence the genes in the gene list. miRNAs with high coefficient indicates that the miRNA highly affect the genes in the gene list directly or indirectly depending on the miRNA-target interaction used. To summarize the regularized regression model, let us assume that GeneSignature represents the list of genes of interest, *miRNetj *is the targets of miRNA (j), and *βj *is the predicted influence coefficient miRNA (j) on the gene signature. The predicted *βj *values are then used to assess the association between miRNAs and gene signatures.

The regularized regression model is described as previously discussed [26]:

(1) $$GeneSignature=\overset{miR}{\underset{j=1}{\sum }}miRNet_{j}*\beta_{j}+\lambda P_{\alpha }\left(\beta \right)$$

where

(2) $$P_{\alpha }\left(\beta \right)=\overset{miR}{\underset{j=2}{\sum }}\left[\frac{1}{2}\left(1-\alpha \right)\beta_{j}^{2}+\alpha |\beta_{j}|\right]$$

is the elastic-net penalty. *α *is a value that ranges from 0 to 1 that penalize correlated variables. When *α *=1, the model is called Lasso regression, and when *α *=0, the model is called ridge regression. Optimizing *α *is critical step to obtain good solution with non-spars regression coefficient values(*β*). Another factor that is important to optimize is *λ *that is critical to shape the sparsity of the solution. Depending on the purpose of the experiment, if there is a large number of variables that need to be reduced (forcing *β *to 0), then *λ *should be set to a high value.

**Figure 1:**
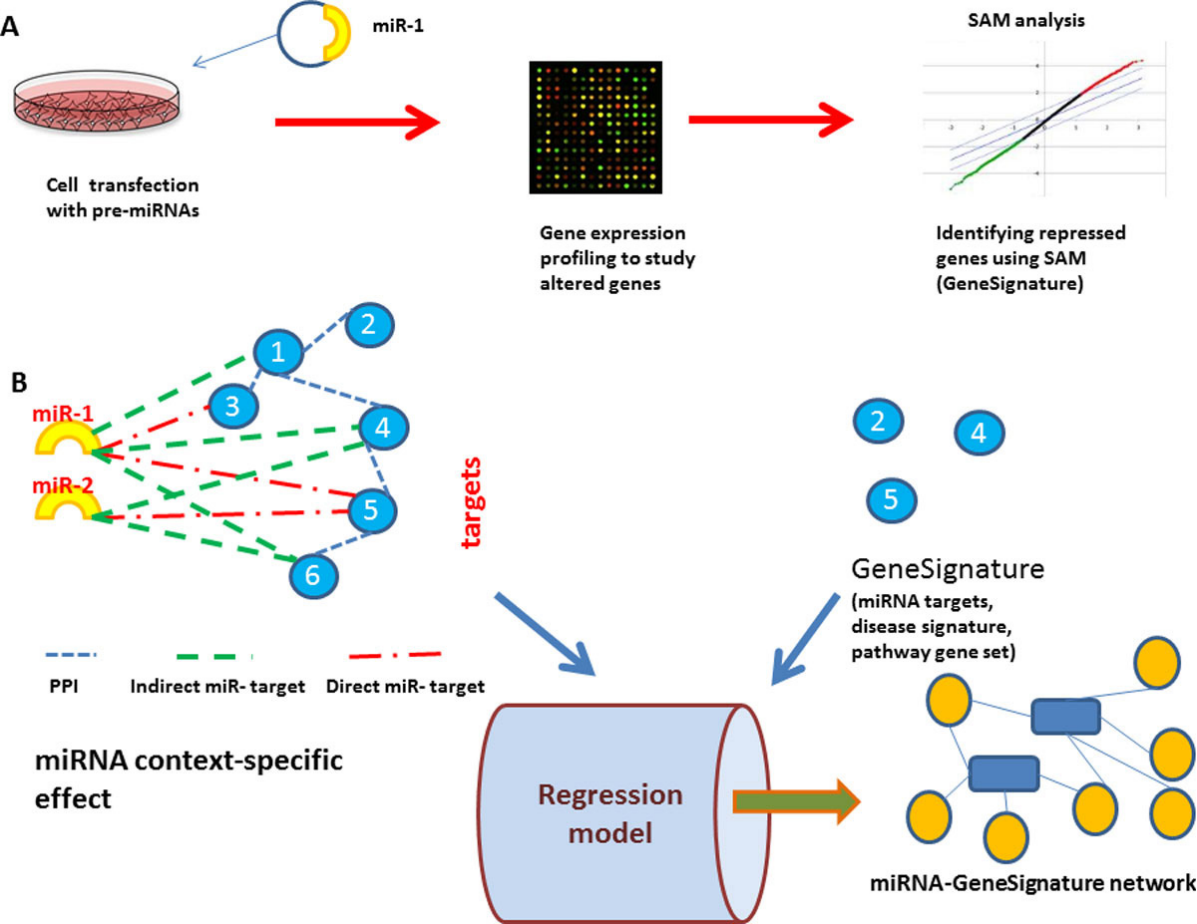
**An overview of constructing influential miRNA-GeneSignature interactions**. A. miRNA gene signature is identified by transfecting cells with pre-miRNA and then identify gene down-regulated upon the transfection. B. Using the context-specific effects of miRNAs (genes affected by miRNAs directly and indirectly) to build functional associations between miRNAs and GeneSignatures via elastic-net regression model. This step sheds light on the functional associations between miRNA and pathways, miRNAs and diseases. It is also used as a miRNA enrichment method to identify miRNAs whose targets are enriched in gene lists. Using miRNA-gene networks and disease or pathway gene networks, the model predicts functional interactions between diseases and miRNAs or pathways and miRNAs.

One of the contributions of this study is to assess the benefit of using the context-specific effect of miRNA for better miRNA functional characterization. We used *miRNet *that includes the context-specific effect of miRNAs as the miRNA signature to predict miRNAs enriched in gene signatures from disease and pathway related genes. We can rewrite the model as:

(3) $$GeneSignature=\overset{miR}{\underset{j=1}{\sum }}\beta_{j}*miRNet_{j}+\lambda P_{\alpha }\left(\beta \right)$$

where *miRNetj *is the targets of *miRNAj *in the context-specific miRNA-target interaction. In this model, *β *is the predicted influence coefficient of miRNAs that represent the enrichment of each miRNA targets in the gene signature. Figure 1 gives an overall description of the major components of the model.

### Context-specific miRNA effect to find functional associations between miRNAs, diseases and pathways

To further validate the applicability of the proposed method to characterize the mode of action of miRNAs, we used curated genes sets from disease and pathway signatures. To build functional association between miRNAs and diseases, and miRNAs and pathways, we extracted disease gene signatures from microarray data related 13 cancers from Gene Expression Omnibus. 450 expression profiles including control and disease samples were extracted to define a gene signature for each disease. All microarray experiments were conducted using GPL96 platform to avoid possible platform bias. In addition to avoid any possible bias that might result from the normalization algorithms, we manually extracted raw data and normalized them using the RMA normalization algorithm [32] implemented in bioconductor. We used Significant Analysis of Microarray (SAM)in order to obtain gene signatures for each disease. For each disease, we only considered the top 200 differentially expressed genes (top upregulated 100 and top downregulated 100) in each experiment. In total, 1942 genes were associated with the 13 cancers. The predicted disease-miRNA interactions of the regression model were validated against a gold standard disease miRNA associations manually extracted from miR2disease [33] and HMDD [34] databases. The gold standard network contains 743 interactions between the 13 cancers and 305 miRNAs. Area Under Curve (AUC) is used to assess the performance of the proposed model and compare it with the other results. On the other hand, to build functional associations between miRNAs and pathways, we used curated pathways from the Molecular Signatures Database (MSigDB) gene sets [18] that contains 1452 canonical pathway gene sets. We removed all pathways that have less than 10 genes and we ended up with 788 pathways. The goal is to find how the miRNA context-specific effect can explain the pathway or disease genes.

## Results

### Parameter optimization

**Figure 2:**
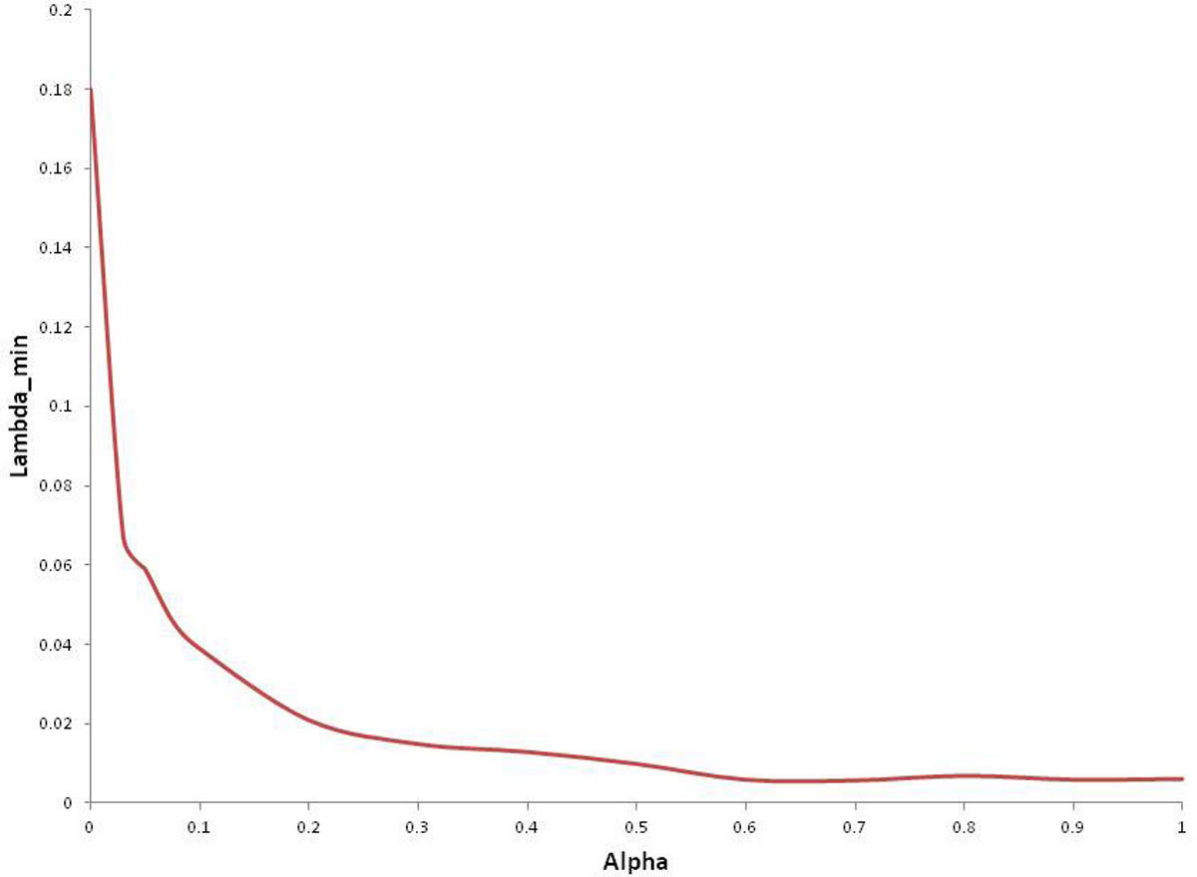
**Optimizing alpha value with respect to min-lambda**. 20 *α *values, ranging from 0 to 1, were initially selected to optimize *α*. For each *α *value, 100 values of *λ *were evaluated. 10-fold cross validation as conducted to select *λ *with minimum meas square error. We selected *α*=0.6 as *λ*-min values started to get steady.

**Figure 3:**
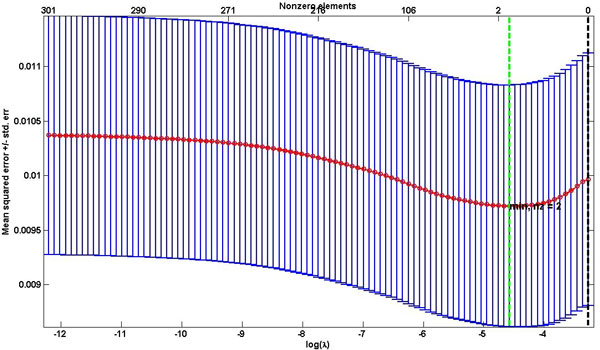
**Mean Square Error vs lambda to optimize lambda value**. Lambda value (*λ*) is optimized using 10-fold cross validation. We selected 100 values of *λ *and used those that minimize the mean square error when *α*=0.6.

In the proposed model, two parameters(*λ *and *α*) that determines sparsity of the solution need to be optimized. As both parameters increase, the number of nonzero influence coefficients (*β*) decreases. In our previous work [26], we described how to optimize both *α *and *λ*. In brief, we selected *α*=0.6 as *λ*-min values started to get steady as shown in Figure 2. For the selected optimal *α *value, 100 values of *λ *were evaluated to select the optimal one (Figure 3) that gives the minimum mean square error. To find regression coefficients, glmnet implementation in MATLAB from http://www-stat.stanford.edu/~tibs/glmnet-matlab was used.

### Regularized regression model identifies the correct miRNA cell treatment

The first line of validation of the effectiveness of the proposed model is to apply it on gene sets of known reference. In this work we assessed the performance of the proposed regression method using several gene lists reported by recently published studies that used expression profiling analysis to discover genes that were downregulated upon miRNA treatment. Full summary of the gene lists used in this study is shown in Table 1. We compiled the list we used in our previous work in addition to a new list of signatures.

To identify the influence coefficient of each miRNA on the gene list of interest (repressed genes after miRNA treatment in this case), the model takes the *miRNet *and the gene signatures. The model output is the coefficient value of each miRNA in *miRNet*. To compare our protein network-based regularized regression model with other ORA based methods, we assessed the performance of each method based on the rank of the miRNA under treatment as specified in Table 1. For example, using the repressed genes after treating LNCaP cells with pre-miRNA-1, all the methods ranked miRNA-1 as the top miRNA. Table 2 summarizes the comparison among the four methods which showed to rank the overexpressed miRNA first (part of this results are taken from our previous work [26]).

**Table 2:** Rank of enriched miRNAs in gene lists downregulated and differentially expressed genes after miRNA treatment

	PPI-based regression model	Regression model	Expression2Kinase	GeneSet2miRNA
*Downregulated gene set in LNCaP cells*				
*pre-miRNA-1*	1*st*	1*st*	1*st*	1*st*
*pre-miRNA-206*	1*st*	1*st*	1*st*	2*nd*
*pre-miRNA-27b*	1*st*	2*nd*	1*st*	1*st*
*pre-miRNA-32*	1*st*	3*nd*	5*st*	3*st*
*pre-miRNA-148a*	1*st*	1*nd*	3*st*	1*st*
*Differentially expressed gene set in LNCaP*				
*pre-miRNA-1 *	1*^st^*	2*nd*	1*^st^*	2*nd*
*pre-miRNA-206 *	1*^st^*	1*^st^*	1*^st^*	2*nd*
*pre-miRNA-27b*	2*nd*	3*rd*	10*th*	15*th*
*Downregulated gene set in HeLa cells*				
*miRNA-1 *	1*^st^*	1*^st^*	1*^st^*	2*nd*
*miRNA-124*	1*^st^*	1*^st^*	2*nd*	2*nd*
*miRNA-373*	1*st*	1*^st^*	2*nd*	2*nd*

The results from this section demonstrate the applicability and effectiveness of the regression models that uses context-specific effect of miRNAs. Our protein-network based regression model outperformed the other ORA based methods (Expression2Kinase and GeneSet2miRNA), and regression model that does not consider protein networks.

### Proposed regression model is robust for gene lists with multiple miRNAs

We next assessed the proposed model on gene sets that are targets of multiple miRNAs. For this test we extracted the targets of 11 prostate cancer miRNAs from PredNet performance of the model on To further apply the proposed model on gene sets that are mixed of multiple miRNA targets, we identified a set of prostate miRNAs that showed to play a role in prostate cancer (Table 3). Only regression models were able to predict the 11 miRNAs as the top 11 retrieved miRNAs (Table 3). This table is adopted from our previous work [26].

**Table 3:** Comparative analysis of four methods to assess their performance to identify the 11 prostate related miRNAs

	PPI-based regression model	regression model	Expression2Kinase	GeneSet2miRNA
*miR-1*	✓	✓	✓	✓
*miR-204*	✓	✓	✓	✓
*miR-143*	✓	✓	✓	✓
*miR-145*	✓	✓	✓	✓
*miR-205*	✓	✓	✓	✓
*miR-221*	✓	✓	✓	
*miR-31*	✓	✓		
*miR-27b*	✓	✓		✓
*Let-7a*	✓			
*miR-133b*	✓	✓		
*miR-222*	✓			✓

✓ indicates that the miRNA is identified in the top 11 enriched miRNAs

### New insights into miRNA systems biology in prostate cancer

**Figure 4:**
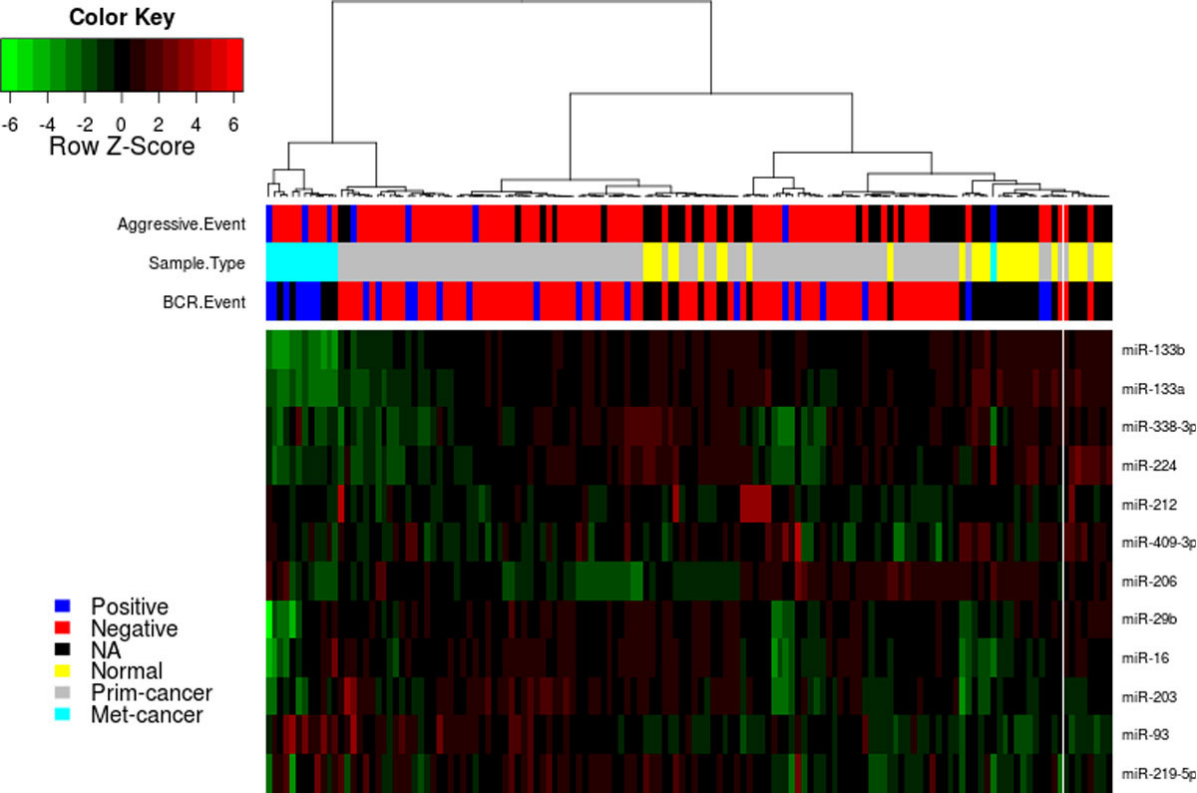
**Heatmap of 12 miRNAs predicted using our model to be enriched in prostate cancer genes**. Using the expression of the 12 miRNAs predicted by our model to be enriched in downregulated genes in prostate cancer, the miRNAs are associated with multiple clinical outcome. This supports our model that it predicts prostate related miRNAs and they can segregate prostate cancer into distinct subtypes.

**Figure 5:**
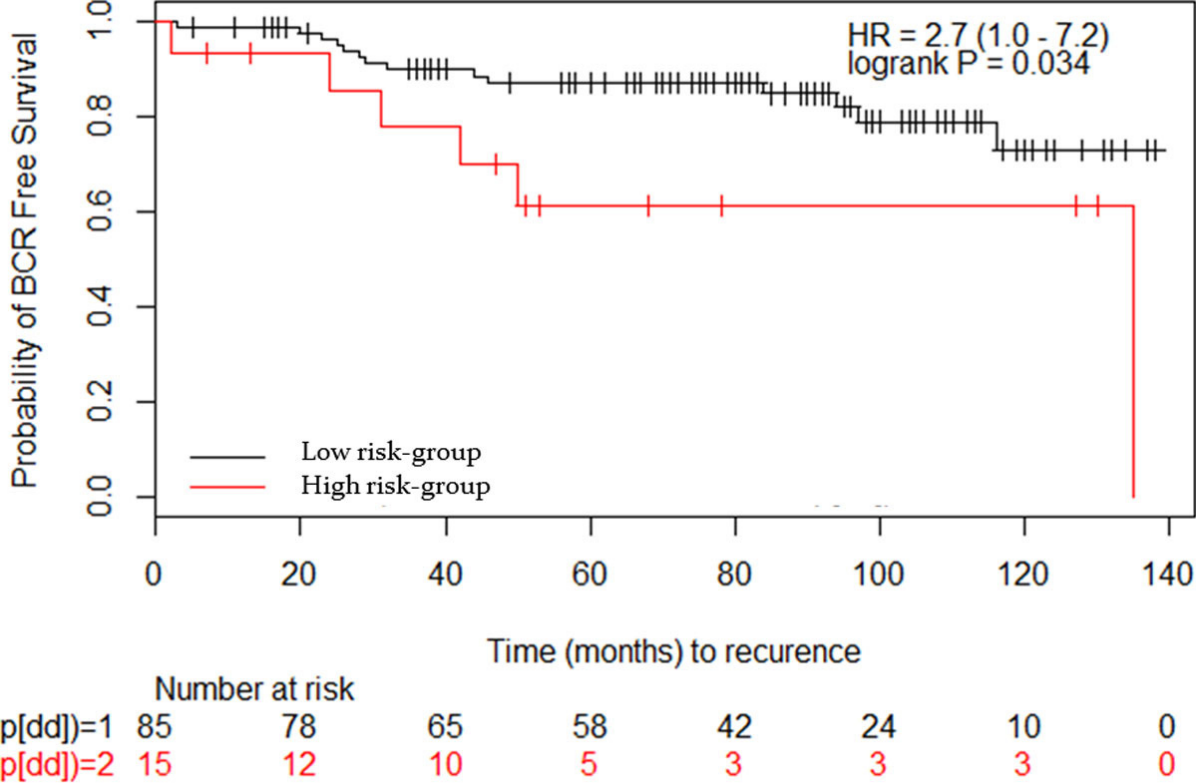
**Kaplan Meier curves of two groups of patients based on BCR related miRNAs**. Using the expression of the 5 miRNAs enriched in BCR related genes, hierarchical clustering was applied to identify two groups and then KM was used to associate them with survival analysis.

The previous analysis conducted in the previous sections provides evidence to the applicability of our model to identify functional associations between miRNAs and gene lists. To take this analysis one step further, we used the 480 downregulated and 51 upregulated (Table 1) genes in prostate cancer to identify miRNAs that gives us more insights on the dysregulated mechanisms in prostate cancer. The proposed model identified 14 miRNAs, 12 of which have expression data in Taylor data(Figure 4). The expression of the 12 miRNAs enriched in the downregulated gene in prostate cancer was extracted from the Taylor data to assess the diagnostic significance in prostate cancer. SVM was able to perform better using the 12 miRNAs predicted by our model (90%) compared to miRNAs from Expression2Kinase(85%). The expression of the 12 miRNAs is associated with cancer recurrence and other clinical variables(Figure 4). Previous experimental work have already shown that miR-146b and miR-206 are prostate cancer related miRNAs and targeting ROCK1 [40] and HDAC4 [36], respectively. Using context-specific miRNA effect regression model, miRNA-16-1 was identified as the most significant miRNA in upregulated genes, in addition to three less significant miRNAs (miR-222, miR-338 and miR-34c). Using Expression2Kinase tool, miR-16-1 did not show significant enrichment. This supports our notion that integrating protein networks to assess overrepresentation of miRNAs in gene lists reveals novel insights to diseases (prostate cancer in this work). Additional analysis was conducted using the miRNAs that were enriched in 269 BCR related genes. Five miRNAs (miR-130a, miR-205, miR-133b,miR-338, miR-429) were predicted to be enriched in the BCR related genes. Using survival (BCR events, time to BCR event) data provided in [39], we build KM curves to assess the biological signal hidden in the five miRNAs. We used hierarchical clustering to group the samples based on the expression of the five miRNAs, and then use KM to assess the significance in the survival data associated with each group. The five miRNAs demonstrated a significant separation between the two groups(Figure 5). This results further supports that protein-based enrichment analysis is a promising direction of enrichment analysis.

### Reconstructing miRNA-disease and miRNA-pathways functional association using miRNA context-specific effect

After demonstrating that elastic-net regression successfully identified miRNAs from downregulated gene lists post to miRNA treatment, we applied the regression modeling to identify miRNAs associated with diseases and pathways using miRNA context-specific effect and disease and pathway signatures. We further analyzed the resulting miRNA-disease and miRNA-pathways functional associations from the regression model.

We first constructed miRNA context-specific effect and gene-disease network to be used as predicted and response variables, respectively, as input to the regression model. miRNA context-specific effect was constructed by integrating results from TargetScan and protein interactions. This study only focused on genes that are targeted by a miRNA and interact with proteins at the protein level. We obtained 3235 genes that are targeted by 305 miRNAs. For the disease gene interactions, we obtained 1942 genes as disease signatures across 13 cancers. Our model generated 741 interactions between the 13 cancers and 305 miRNAs. 364 interactions were common with the gold standard, 157 were in the gold standard and missed by our method, and 220 were identified by the model and not in the gold standard. 37 new interactions were predicted between miRNAs and prostate cancer. Further diagnostic and prognostic characterization of the 37 prostate miRNAs were conducted. We used the 37 miRNAs to evaluate their association with prostate cancer. We extracted the miRNA expression from two prostate cancer data sets. The first is Taylor data [39] (GSE21032) that contains the expression of the miRNAs across 139 samples (98 primary, 12 metastatic and 29 normal). We only obtained 16 miRNAs with expression data in the Taylor data. We first tested the ability of these miRNAs to predict tumor samples. We used support vector machine (SVM) from the LIBSVM library (http://www.csie.ntu.edu.tw/~cjlin/libsvm/) implemented in MATLAB to assess the performance. 10-fold cross validation was used to avoid the overfitting problem. The results show that the newly predicted 16 prostate miRNAs are diagnostically as good as the gold standard prostate miRNAs. The predicted prostate miRNAs were able to classify cancer samples with 90% accuracy in Taylor data. We further conducted survival analysis to assess if the 16 miRNAs are associated with cancer recurrence. The results showed that both the 57 miRNAs in common with the gold standard and the 16 miRNAs predicted are able to significantly separate high risk from low risk patients (p = 0.00025 and 0.007, respectively).

**Figure 6:**
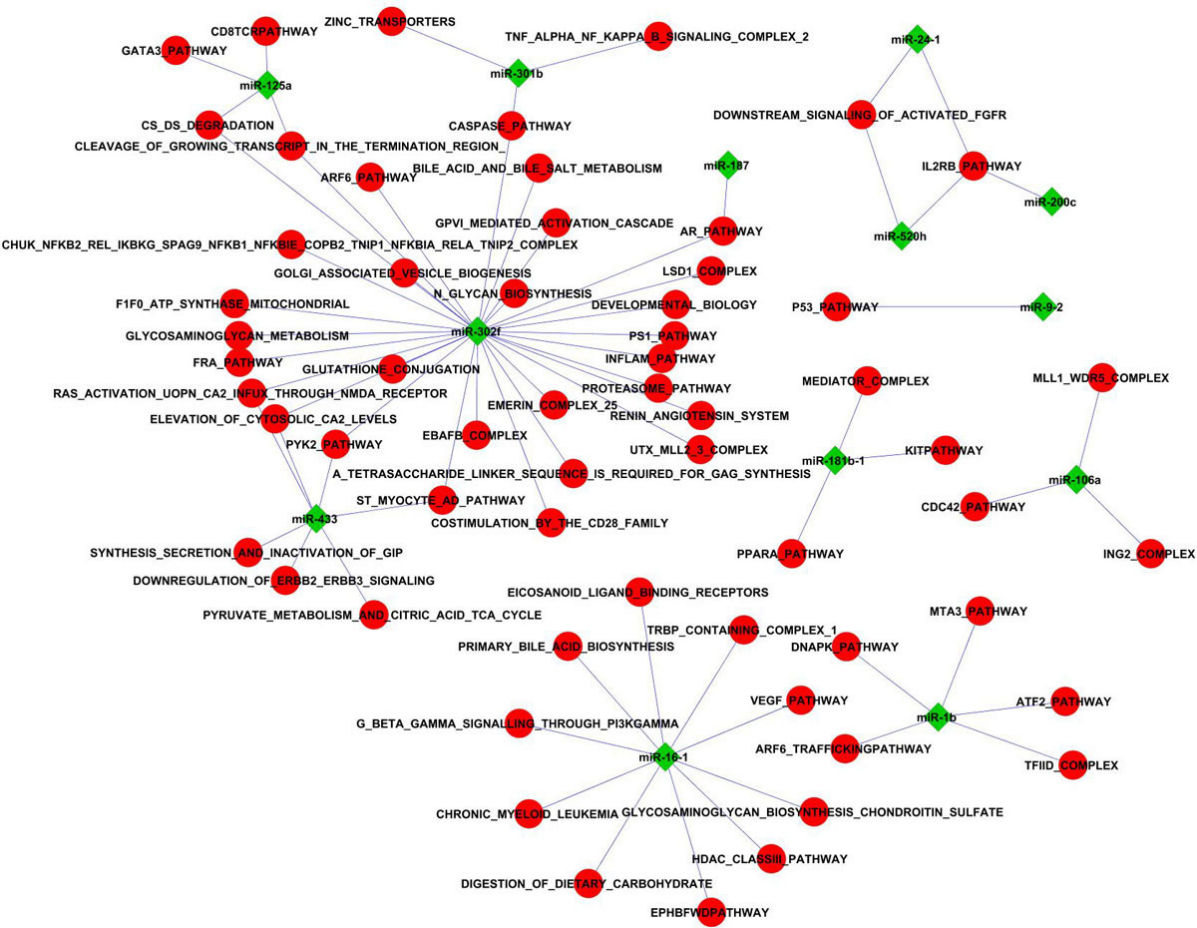
**Functional associations between miRNAs and biological pathways**. Using the context-specific effects of miRNAs and the GeneSignature of pathways as input to the regression model, functional associations between miRNAs and are constructed. In this figure only interactions of regression coefficient greater than 0.5 are selected.

To construct miRNA-pathway functional association, we limited our analysis to the highly significant associations (regression coefficient greater than 0.5) (Figure 6). 77 interactions between 13 miRNAs and 60 pathways. Most of the miRNAs are linked to more than one pathway. miRNA-302f is highly involved in several pathways including Caspase, AR, ARF6 and development pathways. miRNA-1 and 16 are also highly associated with several pathways. We compared this network with miRPath [41], which is a tool that identifies molecular pathways altered by miRNAs. miRPath shows that miR-1b is associated with pentose phosphate and glutathione metabolism pathways, unlike our method that shows it is associated with transcriptional pathways (ATF2, ARF6, DNA-PK). miRPath is unable to associate miR-16-1 to any pathway, however, our method associated miR-16 to several pathways (HDAC, Leukemia, VEGF). This results provides new potential pathways and new miRNA mode of actions that may help to reveal higher level of understanding of miRNA function.

## Discussion

The last decade witnessed a revolution and dramatic changes in high-throughput technologies application in several areas in functional genomics, and are becoming a standard routine in many experimental laboratories. Most these experiments deliver a set of genes relevant to the scientific question under investigation. For example, profiling the gene expression of prostate tumors and normal tissues results in a set of differentially expressed genes that could shed light on the dysregulated pathways in prostate cancer. The first line analysis of gene sets is to reveal the underlying biological knowledge of gene sets. This is accomplished by inferring overrepresentation of curated gene signatures in the gene set of interest. One of the questions asked is what are the regulatory miRNAs that explain a particular gene set. To answer this question, several tools employing gene functional annotations have been developed. These tools assist biologists to characterize the functional role of miRNAs. Most of the developed methods employ statistical overrepresentation analysis like fisher and hypergeometric test. Unfortunately, these methods are static as they do not consider the systematic effect of miRNAs on the protein networks. In this work, we proposed protein network-based regularized regression model to predict influence coefficient of miRNAs on gene list, and thus infer enriched miRNAs in gene sets. High influence here means that the miRNAs are potential regulators for the gene list. Our proposed model is based on miRNA context-specific effect, a miRNA-gene interaction network that considers the indirect association between miRNAs and the targets, to build functional associations between miRNAs and gene signatures.

The first question we asked is that, is context-specific miRNA effect based regression models effective to infer enriched reference molecular concepts(GO terms, disease, pathway, miRNA). We chose to answer this question miRNA gene sets which are list of genes downregulated upon miRNA treatment. Thus we collected several gene sets that were downregulated upon pre-miRNA transfection. Accurate and effective models should infer transfecting miRNAs from the downregulated gene set. The first application of the miRNA context-specific effect is to use it as input to the elastic regression model to predict miRNAs whose targets are enriched in gene lists. Since we know the miRNA that the models should return, we used the rank of the miRNA as a performance assessment measure. Models that rank the correct treatment miRNA are considered as effective and accurate. Based on the results reported in Table 2, the proposed regression model demonstrated a proof-of-concept. Further analyzing the results in Table 2, the methods showed to agree on some cases(miR-1,miR-206) and disagree on others(miR-27b). Most methods prefer gene sets of large size to have good performance.

The proposed model showed to be robust against redundant gene sets. Analyzing the results of the four models on miR-124 and miR-373 revealed that Expression2Kinase and Geneset2miRNA are sensitive to miRNA families. For example, both tools ranked miR-124 and miR-373 second after miR-506 and miR-520 respectively. Looking deeper into the relationships between these miRNAs, we found that the miRNAs predicted second are from the same family of the treating miRNA. For example, miR-1 and miR-206, miR-124 and miR-506, and miR-373 and miR-520 are from the same family and target the same targets.

This is because the elastic net regularize against correlated variables and thus reduce redundant sets. These results demonstrated the effectiveness of the context-specific miRNA effect based elnastic-net regression model as enrichment analysis methodology.

The next step was to apply the model on gene sets with unknown regulatory miRNAs. The objective to identify putative regulatory miRNAs that explains the underlying regulatory mechanism of the gene set. For this test, we used prostate cancer signatures as both miRNA and target expression data are available, in addition to survival and clinical data. Since downregulated genes in prostate cancer can be noisy as they may harbour indirect targets of miRNAs and enriched with multiple miRNAs as there is big body of evidence showing that several miRNAs are dysregulated in prostate cancer. 14 miRNAs were enriched in the genes downregulated in prostate cancer and miR-16-1 was identified to be enriched in the upregulated genes in prostate cancer. The 14 miRNAs are significantly associated with clinical variables of prostate cancer which supports their role in prostate cancer development. Earlier experimental studies [5] showed that miR-16-1 is in clinical trails as a promising prognostic biomarker. Further analysis revealed that miR-16-1 targets BCL2, CCND1, and WNT3A genes [40] which are associated with increased survival and invasion rates. Additional studies [5] support significant role of miR-16-1 in slowing prostate cancer progression, suggesting that using context-specific effect of miRNAs could reveals very significant contribution to the miRNA cancer research. Unfortunately, the expression of miR-16-1 was not available in the miRNA expression data we used and thus we were unable to further confirm its association with clinical outcome.

After showing that context-specific miRNA effect is informative to be used to associate miRNAs with gene signatures, we used it to build functional association between miRNAs and other curated gene sets. In this study we used diseases and pathways curated gene set as response variable in our model with the aim to identify the enriched miRNAs in each gene set. Using gene sets of multiple diseases and pathways, we expected a functional association between miRNAs and the other curated sets. The resulted miRNA-disease associations reveal new associations between miRNAs and diseases, especially prostate cancer. 16 new prostate cancer miRNAs are diganostic and prognostic biomarkers that can be further investigated. The results also uncovered new associations between miRNAs and pathways. Further investigations of the miRNA pathways associations help to explore and validate the power of the model predictions. Our findings here suggest that using protein-network based regularized regression is a new direction of miRNA enrichment analysis that could give us more functional insights into dysregulated pathways or diseases. In addition, the results indicate that miRNA context-specific effect allows defining new mode of action for miRNAs. Using these findings, functional networks associating miRNA with diseases or pathways could be constructed.

## Conclusion

Uncovering miRNA mode of action is a key step to reveal functional associations between miRNAs, diseases and pathways. A crucial task in functional genomics is to interpret gene lists based on curated gene annotations. In this study we used regularized regression model that is trained on novel miRNA-gene interactions network to predict associations between miRNAs and gene sets (diseases, pathways). The model succeeded in the proof-of-concept experiments and showed promise to be applied to other genes lists that harbour biological function. Using the context-specific effect of miRNAs is more effective than just using the direct miRNA targets to infer functional miRNAs from gene lists. The results gained from this study provide higher level of understanding of miRNA function and how it acts as a key regulator molecule in the cellular system. This concludes that the proposed model gives more insight into the functional role of miRNAs in disease development. Although limitations exist in the current work, the uncovered interactions are important for understanding diseases and patterns underlying miRNA-mediated regulations.

## Competing interests

The authors declare that they have no competing interests.

## Authors' contributions

MA designed the study and performed all the analysis. MA wrote the manuscript. RA proof-read the manuscript. All authors read and approved the final version.

## Declaration

The publication costs for this article were funded by the Open Access Fund of The University of Calgary.

This article has been published as part of BMC Bioinformatics Volume 14 Supplement 12, 2013: Selected articles from the IEEE International Conference on Bioinformatics and Biomedicine 2012: Bioinformatics. The full contents of the supplement are available online at http://www.biomedcentral.com/bmcbioinformatics/supplements/14/S12.

## References

[B1] He L Hannon G MicroRNAs: small RNAs with a big role in gene regulation Nat Rev Genet 2004 5 522 531 10.1038/nrg1379 15211354

[B2] Djuranovic S Nahvi A Green R A parsimonious model for gene regulation by miRNAs Science 2011 331 550 553 10.1126/science.1191138 21292970 PMC3955125

[B3] Li L Liu Y Diverse small non-coding RNAs in RNA interference pathways Methods Mol Biol 2011 764 169 182 10.1007/978-1-61779-188-8_11 21748640

[B4] Ruvkun G Molecular biology: Glimpses of a tiny RNA world Science 2001 294 797 799 10.1126/science.1066315 11679654

[B5] Gordanpour A Nam RK Sugar L Seth A MicroRNAs in prostate cancer: from biomarkers to molecularly-based therapeutics Prostate Cancer Prostatic Dis 2012 15 314 319 10.1038/pcan.2012.3 22333688

[B6] Pang Y Young C Yuan H MicroRNAs and prostate cancer Acta Biochim Biophys Sin 2010 42 363 369 10.1093/abbs/gmq038 20539944

[B7] Watahiki A Wang Y Morris J Dennis K ODwyer H Gleave M Gout P Wang Y MicroRNAs associated with metastatic prostate cancer PLoS One 2011 6 e24950 10.1371/journal.pone.0024950 21980368 PMC3184096

[B8] He L Hannon GJ MicroRNAs: small RNAs with a big role in gene regulation Nat Rev Genet 2004 5 522 531 10.1038/nrg1415 15211354

[B9] Calin G Croce C MicroRNA signatures in human cancers Nature Reviews Cancer 2006 6 857 866 10.1038/nrc1997 17060945

[B10] Esquela-Kerscher A Slack F Oncomirs microRNAs with a role in cancer Nature Reviews Cancer 2006 6 259 269 16557279 10.1038/nrc1840

[B11] Trang P Weidhaas J Slack F MicroRNAs as potential cancer therapeutics Oncogene 2008 27 S52 S57 19956180 10.1038/onc.2009.353 PMC10033140

[B12] Ozen M Creighton C Ozdemir M Ittmann M Widespread deregulation of microRNA expression in human prostate cancer Oncogene 2008 27 1788 1793 10.1038/sj.onc.1210809 17891175

[B13] Huang da W Sherman BT Lempicki RA Systematic and integrative analysis of large gene lists using DAVID bioinformatics resources Nat Protoc 2009 4 44 57 19131956 10.1038/nprot.2008.211

[B14] Mostafavi S Ray D Warde-Farley D Grouios C Morris Q GeneMANIA: a real-time multiple association network integration algorithm for predicting gene function Genome Biology 2008 9 S1 S4 18613948 10.1186/gb-2008-9-s1-s4 PMC2447538

[B15] Antonov A Dietmann S Wong P Lutter D Mewes H GeneSet2miRNA: finding the signature of cooperative miRNA activities in the gene lists Nucleic Acids Res 2009 37 W323 W328 10.1093/nar/gkp313 19420064 PMC2703952

[B16] Chen E Xu H Gordonov S Lim M Perkins M Ma'ayan A Expression2Kinases: mRNA profiling linked to multiple upstream regulatory layers Bioinformatics 2012 28 105 111 10.1093/bioinformatics/btr625 22080467 PMC3244772

[B17] Huang D Sherman B Lempicki R Bioinformatics enrichment tools: paths toward the comprehensive functional analysis of large gene lists Nucleic Acids Res 2009 37 1 13 10.1093/nar/gkn923 19033363 PMC2615629

[B18] Subramanian A Tamayo P Mootha V Mukherjee S Ebert B Gillette M Paulovich A Pomeroy S Golub T Lander E Mesirov J Gene set enrichment analysis: A knowledge-based approach for interpreting genome-wide expression profiles PNAS 2005 102 15545 15550 10.1073/pnas.0506580102 16199517 PMC1239896

[B19] Glaab E Baudot A Krasnogor N Schneider R Valencia A EnrichNet: network-based gene set enrichment analysis Bioinformatics 2012 28 451 457 10.1093/bioinformatics/btr678 22962466 PMC3436816

[B20] Stojmirovic A Yu Y Robust and accurate data enrichment statistics via distribution function of sum of weights Bioinformatics 2010 26 2752 2759 10.1093/bioinformatics/btq511 20826881 PMC2958744

[B21] Yip A Horvath S Gene network interconnectedness and the generalized topological overlap measure BMC Bioinformatics 2007 8 22 10.1186/1471-2105-8-22 17250769 PMC1797055

[B22] Poirel C Owens C Murali T Network-based functional enrichment BMC Bioinformatics 2011 12 S14 22479706 10.1186/1471-2105-12-S13-S14 PMC3278830

[B23] Hsu C Juan H Huang H Characterization of microRNA-regulated protein-protein interaction network Proteomics 2008 8 1975 1979 10.1002/pmic.200701004 18491312

[B24] Sualp M Can T Using network context as a filter for miRNA target prediction Biosystems 2011 105 201 209 10.1016/j.biosystems.2011.04.002 21524683

[B25] Sass S Dietmann S Burk U Brabletz S Lutter D Kowarsch A Klaus F Mayer K Brabletz T Ruepp A Theis F Wang Y MicroRNAs coordinately regulate protein complexes BMC Systems Biology 2011 5 136 10.1186/1752-0509-5-136 21867514 PMC3170341

[B26] Alshalalfa M Alhajj R Integrating protein networks for identifying cooperative miRNA activity in disease gene signatures Bioinformatics and Biomedicine (BIBM), 2012 IEEE International Conference on: 4-7 October 2012 2012 1 8 10.1109/BIBM.2012.6392728

[B27] Grimson A Farh K Johnston W Garrett-Engele P Lim L Bartel D MicroRNA Targeting Specificity in Mammals: Determinants beyond Seed Pairing Molecular Cell 2007 27 91 105 10.1016/j.molcel.2007.06.017 17612493 PMC3800283

[B28] Hsu S Lin F Wu W Liang C Huang W Chan W Tsai W Chen G Lee C Chiu C Chien C Wu M Huang C Tsou A Huang H miRTarBase: a database curates experimentally validated microRNA target interactions Nucleic Acids Res 2011 39 D163 D169 10.1093/nar/gkq1107 21071411 PMC3013699

[B29] Xiao F Zuo Z Cai G Kang S Gao X Li T miRecords: an integrated resource for microRNA target interactions Nucleic Acids Res 2009 37 D105 D110 10.1093/nar/gkn851 18996891 PMC2686554

[B30] Wu G Feng X Stein L A human functional protein interaction network and its application to cancer data analysis Genome Biology 2010 11 R53 10.1186/gb-2010-11-5-r53 20482850 PMC2898064

[B31] Tusher V Tibshirani R Chu G Significance analysis of microarrays applied to the ionizing radiation response Proc Natl Acad Sci 2001 98 5116 5121 10.1073/pnas.091062498 11309499 PMC33173

[B32] Irizarry RA Bolstad BM Collin F Cope LM Hobbs B Speed TP Summaries of Affymetrix GeneChip probe level data Nucleic Acids Res 2003 31 e15 10.1093/nar/gng015 12582260 PMC150247

[B33] Jiang Q Wang Y Hao Y Juan L Teng M Zhang X Li M Wang G Liu Y miR2Disease: a manually curated database for microRNA deregulation in human disease Nucleic Acids Res 2009 37 D98 104 10.1093/nar/gkn714 18927107 PMC2686559

[B34] Lu M Zhang Q Deng M Miao J Guo Y Gao W Cui Q An analysis of human microRNA and disease associations PLoS One 2008 3 e3420 10.1371/journal.pone.0003420 18923704 PMC2559869

[B35] Lim L Lau N Garrett-Engele P Grimson A Schelter J Castle J Bartel D Linsley P Johnson J Microarray analysis shows that some microRNAs downregulate large numbers of target mRNAs Nature 2005 433 769 773 10.1038/nature03315 15685193

[B36] Hudson RS Yi M Esposito D Watkins SK Hurwitz AA Yfantis HG Lee DH Borin JF Naslund MJ Alexander RB Dorsey TH Stephens RM Croce CM Ambs S MicroRNA-1 is a candidate tumor suppressor and prognostic marker in human prostate cancer Nucleic Acids Res 2012 40 3689 3703 10.1093/nar/gkr1222 22210864 PMC3333883

[B37] Jalava S Urbanucci A Latonen L Waltering K Sahu B Jnne O Seppl J Lhdesmki H Tammela T Visakorpi T Androgen-regulated miR-32 targets BTG2 and is overexpressed in castration-resistant prostate cancer Oncogene 2012 31 4460 71 10.1038/onc.2011.624 22266859

[B38] Porkka K Pfeiffer M Waltering K Vessella R Tammela T Visakorpi T MicroRNA Expression Profiling in Prostate Cancer Cancer Research 2007 67 6130 10.1158/0008-5472.CAN-07-0533 17616669

[B39] Taylor B Schultz N Hieronymus H Gopalan A Xiao Y Carver B Arora V Kaushik P Cerami E Reva B Antipin Y Mitsiades N Landers T Dolgalev I Major J Wilson M Socci N Lash A Heguy A Eastham J Scher H Reuter V Scardino P Sander C Sawyers C Gerald W Integrative Genomic Profiling of Human Prostate Cancer Cancer Cell 2010 18 11 22 10.1016/j.ccr.2010.05.026 20579941 PMC3198787

[B40] Saini S Majid S Dahiya R Diet, microRNAs and prostate cancer Pharm Res 2010 27 1014 1026 10.1007/s11095-010-0086-x 20221895 PMC2872011

[B41] Vlachos I Kostoulas N Vergoulis T Georgakilas G Reczko M Maragkakis M Paraskevopoulou M Prionidis K Dalamagas T Hatzigeorgiou A DIANA miRPath v.2.0: investigating the combinatorial effect of microRNAs in pathways Nucleic Acids Res 2012 40 W498 504 10.1093/nar/gks494 22649059 PMC3394305

